# The impact of additive or substitutive clinical study design on the negotiated reimbursement for oncology pharmaceuticals after early benefit assessment in Germany

**DOI:** 10.1186/s13561-020-00263-2

**Published:** 2020-03-14

**Authors:** C. M. Dintsios, I. Beinhauer

**Affiliations:** 1 Medical Faculty, Institute for Health Services Research and Health Economics, Heinrich Heine University, Building: 12.49, Moorenstr. 5, 40225 Düsseldorf, Germany; 2 Health Economics, Cologne, Trainee at Bayer Vital GmbH, Leverkusen, Germany

**Keywords:** Study design, Negotiated prices, Oncological drugs, Pharmaceutical companies, Payers, German statutory health insurance, I11, I13, I18

## Abstract

**Background:**

We analysed the impact of clinical study design for oncological pharmaceuticals on the subsequent price negotiations after early benefit assessment between pharmaceutical companies and the German National Association of Statutory Health Insurance Funds. The analysis was conducted for all oncology pharmaceuticals that underwent the early benefit assessment in Germany since its introduction in 2011 up to September 2016.

**Methods:**

It was differentiated between additive (new therapy in addition to baseline therapy) and substitutive study designs (baseline therapy to be replaced). The study design was derived from the dossiers of the pharmaceutical companies submitted to the Federal Joint Committee. Subgroup specific costs in case of granted added benefit were calculated as annual therapy costs and compared with the costs of the appropriate comparators to quantify price premiums. Further price influencing factors were analysed in univariate and multivariate regression analysis considering the budget impact for the statutory health insurance as well.

**Results:**

The mean and the median of the additive premiums for substitutive designs (€50,477.68 and €49,841.24) were higher than for additive designs, if the comparator was different to best supportive care (€48,750.00 and €42,820.44). The mean multiplicative premium for the substitutive designs was 15.07 versus 2.29 for the additive designs. EU-Prices and target population size had a significant effect on the reimbursement. The adjusted R-square in the log Premium OLS-regressions reached 0.708 when including all explanatory variables and considering interaction between target population and annual costs of the comparator.

**Conclusions:**

Study design as an additional important influencing factor of the negotiations next to those stated in the framework agreement was identified and verified. Therefore, study design should be considered by pharmaceutical companies and by decision makers and payers within strategic price planning as a potential predictor. For some specific categories the number of cases was small. Further analyses should be performed when more oncology pharmaceuticals have passed the early benefit assessment.

## Introduction

### Statutory framework

Predicted Statutory health Insurance (SHI) deficits for 2010 and 2011, of 7 billion € and 10–12 billion €, respectively, resulted in a law freezing the prices of pharmaceuticals already in the market (which came into effect on 1 August 2010) and subsequently the German parliament passed the ‘*Act to Reorganize the Pharmaceutical Market in the Statutory Health Insurance System*’ (AMNOG) on 11 November 2010 [1]. AMNOG came into effect on 1 January 2011 and brought about a *paradigm shift* for the market access of pharmaceutical innovations in Germany. Even beforehand, a number of legal reforms with approaches of cost containment were introduced but none of them had the intention of implementing a benefit assessment of pharmaceuticals [2]. Now with the introduction of the AMNOG, an *added benefit* of new pharmaceuticals based on patient-relevant outcomes (mortality and patient reported morbidity or safety inclusively quality of life, but no surrogates unless validated according to strict methodological rules) has to be demonstrated against an *appropriate comparative therapy* (ACT). This ACT is based on the principles of evidence-based medicine in accordance with the German Social Code V (Paragraph 35a, Section 1, Sentence 8) and serves the time-shifted reimbursement negotiations by means of an early benefit assessment (EBA), as in the first year after launch the price is set by the manufacturer.

The day of the market entrance of a newly authorized pharmaceutical marks the start of the EBA. Pharmaceutical manufacturers have to submit a benefit dossier according to a formalistic template to the *Federal Joint Committee* (FJC; in German Gemeinsamer Bundesausschuss: G-BA). Prior to that, they can participate optionally in a scientific advice offered by the FJC [3]. The FJC is the German *self-administrative body* of physicians, dentists, hospitals, and statutory health insurance (SHI) funds. It effectuates the framework provided by the legislation and ensures that legal instructions are implemented in the healthcare system. The regulations issued by the FJC represent *binding sublegal norms*, which apply to the SHI funds, the insured persons, physicians, and other service providers. Areas covered are: prescription of medicines, national needs-planning for specialist practices, assessment of examinations, treatment methods in outpatient and inpatient care, services ordered by doctors, and disease management programs [4]. The FJC commissions according to the German Social Code V (Paragraph 139b, Section 1) the *Institute for Quality and Efficiency in Health Care* (IQWiG), which was established as a professionally independent, supporting scientific institute. The IQWiG primarily prepares *evidence reports* on pharmaceuticals and non-drug interventions, and assesses the EBA dossiers of new pharmaceuticals. The methodological basis of its benefit assessment and uncertainties regarding outcome and study results are covered in IQWiG’s publication on ‘General Methods’ [5] and some specific publications [6, 7].^1^

Within 3 months of submission, the dossier is evaluated in most cases by the IQWiG. The dossier must include data from all studies that meet the inclusion criteria (e.g. all relevant studies against the chosen ACT); the other studies (e.g. intervention not in line with label etc. are just listed and excluded from the assessment), as well as information on the approved indication, benefit, added benefit in comparison with appropriate therapeutic alternatives, costs of treatment, number of patients and patient groups experiencing a therapeutically relevant added benefit, and any special requirements to ensure appropriate use of the new drug and the predefined comparator. Details on the dossier are further specified in the *Legislative Decree on the benefit assessment of pharmaceuticals* (in German Arzneimittel-Nutzenbewertungsverordnung: AM-NutzenV) [12] issued by the Ministry of Health and in the FJC’s rules of procedure (in German: Verfahrensordnung des Gemeinsamen Bundesausschusses: G-BA VerfO) [13]. The IQWiG evaluation results in a *recommendation* regarding the added patient-relevant benefit of the investigated drug (assessment).

A hearing is established with regard to submitted comments on IQWiG’s evidence report by entitled stakeholders in between the time of recommendation by IQWiG and the time of the final decision by the FJC. Addenda can be commissioned by the FJC as a result of the hearing or in cases in which the need for additional work arises during the course of consultations. Addenda offer supplementary information provided at short notice by IQWiG on respective issues.

Three months after IQWiG’s recommendation, the FJC concludes the benefit assessment by making a *final decision* regarding the added benefit (appraisal). For orphan drugs, both, assessment and appraisal regarding clinical evidence are performed by the FJC. Their market authorisation is considered proof of added benefit, which has yet to be quantified by the FJC, but only up to annual revenue of 50 million Euros. Once this sales threshold is exceeded, orphan drugs are assessed as conventional drugs. The FJC’s decision is based on the manufacturer’s dossier, IQWiG evaluation, as well as the results of the submitted comments and subsequent public hearing and addenda, if any.

### Benefit assessment

Outcomes considered by IQWiG and FJC in terms of added benefit are grouped into three dimensions: *mortality, morbidity* including (severe) adverse events, and *health-related quality of life* (HRQoL). Fewer *adverse events* in comparison to the ACT are considered as an added benefit of the assessed pharmaceutical. All relevant available information from clinical studies on adverse events have to be included in the dossier [5, 13].

In the case of an acknowledged added benefit this benefit can vary in different extents (major, considerable, and minor or in the case of a not determinable added benefit: not quantifiable). Further, the benefit can also be classified as not available (no added benefit) or lesser in comparison to the ACT. The FJC determines the ACT and additional *subgroups* (SG) for assessment [13]. However, it assesses and acts based on the same value dimensions (desirable effect [benefit and value] and harm [side effects and risks]) as *market authorisation* [14, 15].^2^ According to the *rules of procedure* of the FJC, the criteria for determining the ACT are: (i) If a medicinal product is considered as the comparator, it must be approved for the respective therapeutic indication. (ii) If a non-pharmaceutical treatment is considered as the comparator, this must be deliverable within the framework of the SHI. (iii) Pharmaceuticals or non-pharmaceutical treatments whose patient-relevant benefit has already been determined by the FJC are preferred as comparator. (iv) The comparator should belong to the appropriate therapy in the therapeutic indication according to the generally accepted state of medical knowledge. (v) If there are several alternatives, the most economical therapy is selected, preferably a therapy, for which there is a reference price (this criterion applies to price negotiations only). Since 2013, the *option for multiple comparators* has been regulated by law. This option allows the manufacturer if FJC has set a list of equal ACTs to choose one ACT out of these ACTs.

In addition to the extent of added benefit versus the ACT the quality of the *evidence base* is evaluated. The evidence level is rated as proof, indication, or hint on the basis of the number and characteristics of the submitted studies, the certainty of the results, and the consistency of the observed treatment effects [5]. The highest evidence level requires a statistically significant effect in a meta-analysis or at least two independent randomized controlled trials showing statistically significant treatment effects in the same direction. Lower evidence levels are assigned when the presented evidence is based on only one randomized controlled trial or is considered to have a higher potential for bias [5].

### Price negotiations

After the FJC decision, *price negotiations* between the National Association of SHI Funds (GKV SV) and the manufacturer on the reimbursement amount begin. These negotiations are based on the *framework agreement*, signed by the National Association of SHI Funds and the four relevant pharmaceutical companies’ unions (vfa, BPI, BAH, and ProGenerika). The main points to consider within the negotiations according to the framework agreement [18] are: (i) the *annual therapy costs* (AnTC) of the *ACT*, defined by the FJC, (ii) the *extent of the added benefit* as a result of the early benefit assessment, expressed by the respective categories together with the uncertainty of the submitted evidence (i.e. the evidence level), (iii) *comparable pharmaceuticals* within the authorized indication(s) of the assessed drug, and (iv) *European prices* in the referenced countries adjusted at *purchase power parity* and weighted by the respective sales volumes [19].^3^ The European countries, which are looked at while comparing the prices, are included in a specific basket of countries. This basket includes the following countries: Belgium, Denmark, Finland, France, Greece, United Kingdom, Ireland, Italy, the Netherlands, Austria, Portugal, Sweden, Slovakia, Spain, and the Czech Republic.^4^ The choice of countries was based on three criteria: (i) countries from all states of the European economic area, (ii) countries with an additive population of 80% of the European economic area (excluding Germany), and (iii) countries with a similar economic performance compared with Germany.

The framework agreement clarifies that the negotiations follow a *premium pricing* philosophy in the sense of a mark-up calculation on the AnTC of the ACT (i.e. exactly the opposite of rebates). Furthermore, the reimbursement amounts are derived by taking into account every *subpopulation* particularly [20, 21]. Thus, the implemented approach could be characterized as a mixed-calculation-approach, using *prevalence* data to weight the respective partial reimbursement amount for each subpopulation.

Whereas an added benefit supports a negotiable price premium over the ACT, pharmaceuticals that are not granted an added benefit by the FJC are assigned to a *reference price group* if possible or priced with the price of the ACT as an upper limit. The price negotiations must be finalized within 6 months after the decision. If no agreement is reached during this time, an *arbitration board* is called [22].

Subsequently, both contracting parties can request a *cost-benefit assessment* at the FJC, which is again conducted by the IQWIG. Moreover, they can take *legal actions*, but neither cost-benefit assessment nor the legal actions have delaying effects. Thus, the reimbursement amount set is valid from an agreed date during negotiations or a date determined by arbitration. Up to now, no cost-benefit assessment has been applied for by any contracting party.

**Fig. 1:**
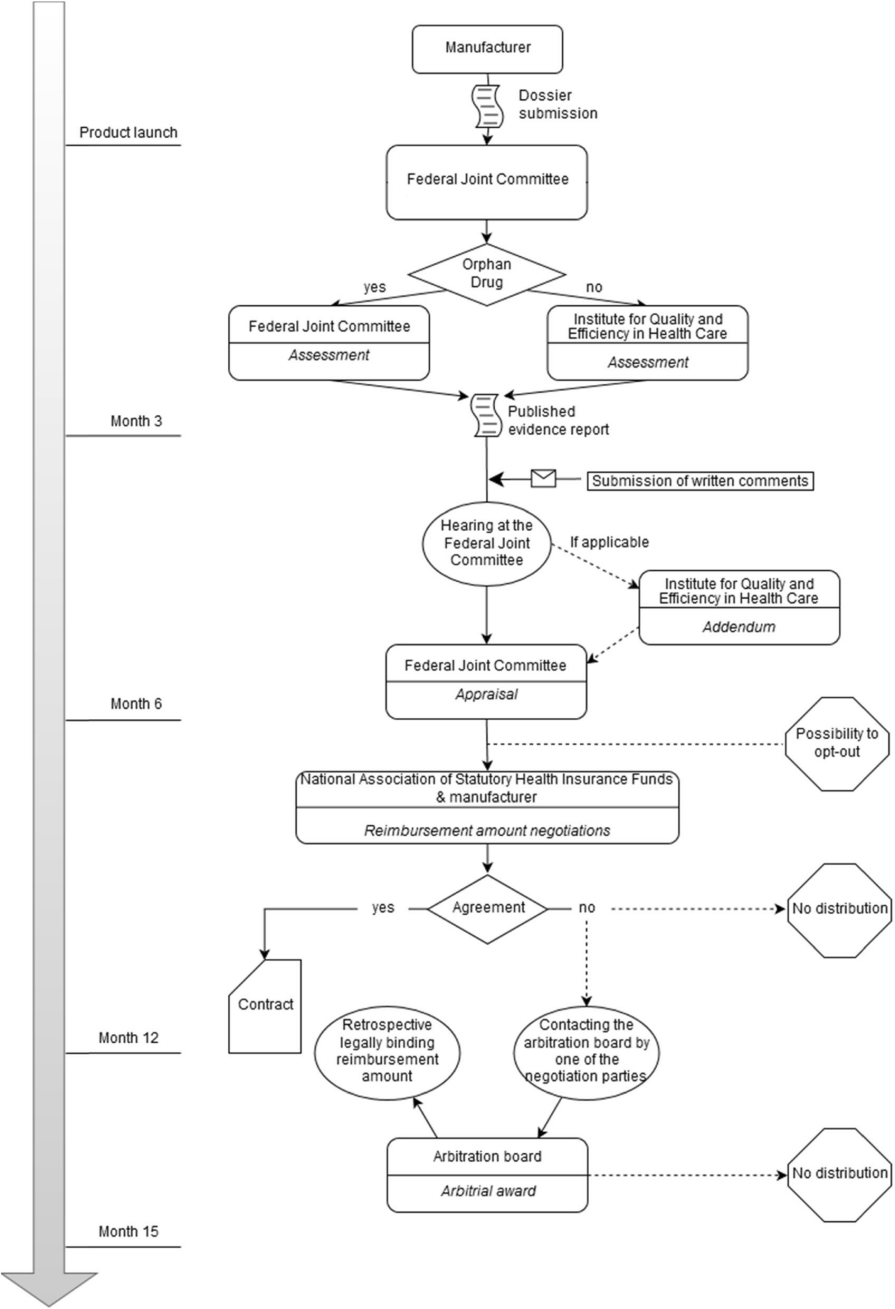
Complete AMNOG process. The figure depicts the complete AMNOG process in all its steps

The complete process from early advice and dossier submission to the point of price negotiations and subsequent arbitration, if necessary, is delineated in Fig. 1.

### Study design variability

The design of clinical trials included in the dossier submission of pharmaceutical companies to the FJC can vary, irrespective of its evidence level (i.e. (randomized) controlled trials, case-control studies, narrative comparisons with historical controls etc.). In general, there are *additive study designs* adding to the baseline therapy (comparator arm) a new therapy (intervention arm) (e.g. Intervention + Best Supportive Care versus Placebo + Best Supportive Care), and *substitutive study designs* replacing in the intervention arm the therapy of the control arm with the new therapy, (e.g. Intervention versus Best supportive Care). In the present analysis, the negotiated reimbursement amounts are differentiated by the study design and the impact of the latter as such on the reimbursement amount is examined. The analysis is performed especially for oncological products, since in this therapeutic field different clinical study designs are common. In oncology, new therapies are implemented frequently in progressed therapeutic lines and therefore in addition to already existent therapies. Hence, additive study designs are often chosen for the clinical testing. On the other hand, substitutive study designs are used for clinical testing in oncology as well. Furthermore, many oncological products are approved with an *orphan drug designation*. Orphan drugs can be differentiated in such being absolute *soloists*, meaning that no other pharmaceuticals existed in the orphan disease indication before their market authorisation and early benefit assessment, and in orphan drugs competing with other existing orphan drugs in the same orphan disease indication, respectively. For orphan drugs, a differentiation of the clinical studies with additive or substitutive design is possible as well.

### Hypothesis testing

The study design determines the therapeutic regime and, thus, it influences directly the *budget impact* of a pharmaceutical for the SHI. An additive study design is accompanied by a higher budget impact, since additional costs for the new therapy accrue on top of the baseline therapy. In case of a substitutive study design the costs for the substituted baseline therapy cease and, thereby, the budget impact becomes lower than in case of an additive study design. The budget impact of a new pharmaceutical is always considered in the price negotiations within AMNOG, since the main purpose of AMNOG was cost containment of pharmaceutical expenditure for the SHI. The primary hypothesis to be tested is:

- *The design of the submitted studies within early benefit assessment has an impact on the negotiated reimbursement amounts*.

To test this primary hypothesis, *secondary hypotheses* with regard to the impact on price negotiations of different study designs and a proven or unproven added benefit are derived. This is done by analysing the premium on the AnTC of the ACT according to the framework agreement between the National Association of the SHI and the pharmaceutical companies’ unions.

1. AnTC of new therapy = AnTC of ACT + premium

#### Hypothesis 1

For additive study designs and no proven added benefit, the AnTC of the therapy are not exceeding the AnTC of the ACT. It is expected that the price for the new therapy will be zero, since the AnTC for the baseline therapy (e.g. Best Supportive Care) are crossed out. In particular cases even negative reimbursement amounts could be theoretically expected when taking into account for the AnTC additional diagnostic or other measures with regard to the intake of the new drug, contained in the package leaflet.

#### Hypothesis 2

For substitutive study designs and no proven added benefit, the AnTC of the new therapy *do not exceed* the AnTC of the ACT.

#### Hypothesis 3

For additive study designs and a proven added benefit, a *premium* on the AnTC of the ACT is *negotiated* (which is lower than the premium for substitutive study designs as the higher budget impact of combination therapies with additive study design is offset by lower premiums).

#### Hypothesis 4

For a substitutive study design and a proven added benefit, a *premium* on the AnTC of the ACT is *negotiated* (which is in turn of hypothesis 3 higher than in case of additive study designs).

#### Hypothesis 5

Depending on the study design, analogous scenarios as in hypotheses 3 and 4 can be derived for orphan drugs, on the premise that no ACT is defined for orphan drugs and, therefore, the *reference price* for the price negotiations is set by the *comparable pharmaceuticals*, if any, in the orphan indication. In case of absolute orphan drug *soloists*, no AnTC of comparable pharmaceuticals are available to negotiate a premium on their basis; the negotiated reimbursement amount reflects then the direct SHI *willingness to pay* for the new therapy.

**Fig. 2:**
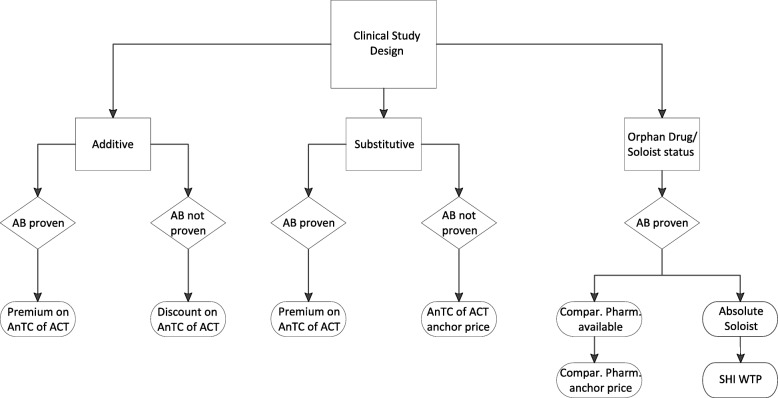
Hypotheses to be tested. The figure visualizes the hypotheses to be tested according to the study design of the submitted evidence for the oncology products for which an added benefit was granted by the Federal Joint Committee and a negotiated reimbursement amount is available in the period under consideration

Figure 2 visualises the secondary hypotheses.

## Methods

The last 6 years, beginning with the implementation of the AMNOG in the SHI system up to end of 2016, are offering certain evidence on the study design and its impact next to other factors on the reimbursement price negotiations. Hence, within the given negotiated prices for oncology products, every case was studied, critically reviewed and analysed. In order to do so, we proceeded with a *multistage approach* comprising five steps:

1. Based on the decision of the FJC we extracted the following *data*: (i) pharmaceutical company, (ii) approved indication, (iii) subgroup-specific patient population size (in case a range is only given, we used the mean), (iv) therapeutic intervention, (v) appropriate comparative therapy, (vi) added benefit and evidence level, (vii) date of the decision, (viii) annual therapeutic costs of the appropriate comparative therapy, (ix) annual consumption of the oncological pharmaceuticals, and (x) costs for additional SHI services, if any. If for individual cases with regard to subgroups or the total target populations no studies were submitted or IQWiG considered the studies being not appropriate, these *cases* were *excluded* from our analysis. To check, if included orphan drugs were absolute soloist or if further competitors were available in the German market for the labelled indication, we used the classification system by Fricke [23]. This classification divides pharmaceuticals depending on their innovation level into four classes. The first class includes pharmaceuticals with a complete new molecular structure or mode of action and, therefore, no competition in their indication area. The third class refers to me-too pharmaceuticals or such with marginal modifications. Hence, Orphan drugs classified at the first class were considered as absolute soloists; those classified in the third class were considered as competitive pharmaceuticals, respectively. To validate these *classifications* we crosschecked module one of the submitted dossiers by the pharmaceutical companies searching for comparable pharmaceuticals.
2. In addition to the information derived from the FJC decisions, the AnTC before and after the negotiations for each dosage and package referred to in the FJC decisions were calculated from the price information given in the German Drug Directory (Lauer-Taxe) in step 2.^5^ For this purpose we used the following formula:
3. Annual package consumption x net costs per package (after subtraction of the mandatory SHI rebates)^6^ + costs for necessary additional SHI services + costs for other SHI services = AnTC of the therapy^7^
  In case a range for different dosages is given in the decision of the FJC, the *mean* was used.
4. In the next step, *premiums* on the AnTC of the comparative therapy were calculated according to (i) an *additive* and to a (ii) *multiplicative approach*, respectively. We used the following formulas:
5. Additive premium on subgroup-specific ACT:
  AnTC after negotiation = SG_1_ x (AnTC ACT_1_ + Premium_1_) + SG_2_ x (AnTC ACT_2_ + Premium_2_).

All cases included in the analysis had no more than 2 subgroups. SG is used here as the normalized weight of each subgroup size among the total target population of the appraised drug.

(3) Multiplicative premium on subgroup-specific ACT
  AnTC after negotiation = SG_1_ x AnTC ACT_1_ x Premium_1_ + SG_2_ x AnTC ACT_2_ x Premium_2_
(4) Additive premium on weighted ACT:
  AnTC after negotiation = (SG_1_ x AnTC ACT_1_ + SG_2_ x AnTC ACT_2_) + Premium
(5) Multiplicative premium on weighted ACT:
  AnTC after negotiation = (SG_1_ x AnTC ACT_1_ + SG_2_ x AnTC ACT_2_) x Premium.

The *subgroup-specific premiums* are the basis for our analysis with regard to the implemented study design, since the study design itself and the extent of added benefit may vary across different subgroups. The subgroup-specific premiums reflect the monetized added benefit for each subgroup, respectively. They were subsequently derived from the above mentioned formulas by conversion (e.g. for a subgroup-specific multiplicative premium assuming no added benefit for subgroup 2 and an added benefit for subgroup 1):

(6) Premium SG_1_ = (AnTC after negotiation – SG_2_ x AnTC ACT_2_)/SG_1_ x AnTC ACT_1_

For the estimation of the *budget impact,* the additive premiums on a weighted ACT are useful. The additive premium on a weighted ACT estimates the direct total extra expenses from a *SHI perspective* arising from the market entrance of a new therapeutic intervention authorized in more than one patient group or indication. We implemented an additive and a multiplicative calculation approach of the subgroup-specific premiums in general, because both approaches are valuable. The additive premiums are easier to interpret representing absolute price differences in monetary units (€). The multiplicative premiums are more advantageous for a relative analysis when comparing cases with low and high priced ACT. For cases with one subgroup with and another without added benefit, we assumed according to the German Social Code^8^ that for the subgroups without added benefit no premiums on the AnTC of the subgroup-specific ACT are negotiated. This translates for the additive premiums into 0 € and for the multiplicative premiums in a multiplier of 1. In Table 1 the calculation examples for the premium calculation of Axitinib are presented.

**Table 1:** Premium calculation for Axitinib

1. Estimation of AnTC of the appraised pharmaceutical after negotiation:	
Average annual tablet consumption according to the decision of the FJC: 730–1460	
Package size: 56 tablets	
Annual package consumption: [(730 + 1460)/2]56 = 19.5536	
Additional SHI services or other SHI costs: 0 €	
Date of FJC decisdion: March 2013 ➔ search for price adaptation in German Drug Directory (Lauer) September/October 2013	
Date of new price for Inlyta 5 mg 56 tabl.: 01.10.2013	
Pharmacy selling price (PSP): 5596.87 €	
Mandatory pharmacy rebate: 1.85 €	
Mandatory manufacturer rebate: 1941.53 €	
Costs after subtraction of statutory mandated rebates: 5596.87–1.85 – 1941.53 = 3653.49 €	
AnTC after negotiation = 3653.49 × 19.5536 = 71,438.88 €	
2. Estimation of AnTC of the appraised before negotiation:	
To estimate costs of Inlyta 5 mg 56 tabl. Before negotiation all the data were extracted from the German Drug Directory (Lauer) the 15th September 2013, so that no bias due to changed statutory manufacturer rebates occur:	
PSP: 5596.87 €	
Mandatory pharmacy rebate: 1.85 €	
Mandatory manufacturer rebate: 723.12 €	
Costs after subtraction of statutory mandated rebates: 5596.87 € - 1.85 € - 723.12 € = 4871.90 €	
AnTC before negotiation = 4871.90 € × 19.5536 = 95,263.19 €	
3. AnTC of the ACT (added benefit):	
Subgroup 1: Everolimus 49,569.47 €; No added benefit	
Subgroup 2: Sorafenib 55,314.19 €; Indication for a minor added benefit	
4. Estimation of subgroup proportion:	
Subgroup 1: 914 patients = 914/920 = 0.99 weighting coefficient SG_1_	
Subgroup 2: 6/920 patients = 0.01 weighting coefficient SG_2_	
5. Calculation of premiums	
5.1 Additive premium on subgroup-specific ACT:	
Premium_2_ = (71,438.88 € - 0.99 × 49,569.47 € - 0.01 × 55,314.19 €)/0.01 = 2,181,196.49 €	
5.2M ultiplicative premium on subgroup-specific ACT:	
Premium_2_ = (71,438.88 € - (0.99 × 49,569.47 €))/(0.01 × 55,314.19 €) = 40,432.85 €	
5.3 Additive premium on weighted ACT	
Premium 71,438.88 € - 0.99 × 49,569.47 € - 0.01 × 55,314.19 € = 21,811.96 €	
5.4 Multiplicative premium on weighted ACT	
71,438.88 €/(0.99 × 49,569.47 € + 0.01 × 55,314.19 €) = 1.4395	

In case of pharmaceuticals with a *market authorization* for *more than one indication,*^9^ the premiums for the *first indication* can be directly considered in the analysis, whereas for the *second indication* the analysis has to be more sophisticated to avoid any bias due to the premium calculation for the first indication. Two approaches can be implemented for the premium of the second indication: (i) a *mixed calculation* for the premium or (ii) the calculation of a *partial reimbursement amount*. For the first, the study design is not considered. In case that the indications refer to different study designs; only a conclusion on the overall premium is possible and a third mixed category comprised of additive and substitutive study designs has to be composed. The following formula refers to the case of two indications in which the second one consists of two subgroups:

(7) (Proportion Ind_1_ x AnTC ACT_1_) x Premium + Proportion Ind_2_ x (SG_1_ x AnTC ACT_SG1_ x Premium + SG_2_ x AnTC ACT_SG2_ x Premium) = Proportion Ind_1_ x Consumption Ind_1_ x Price after negotiation + Proportion Ind_2_ x Consumption Ind_2_ x Price after negotiation

The partial reimbursement amount approach considers the study design of the different indications. Initially, the actual reimbursement amount is subdivided depending on the prevalence of each indication. The reimbursement amount for the first indication is given by the results of the first negotiation. Thus, the hypothetical reimbursement amount for the second indication can be likewise calculated:

(8) Prevalence Ind_1_ x Reimbursement amount_1_ + Prevalence Ind_2_ x Reimbursement amount_2_ = actual reimbursement amount

Even if the reimbursement amount for the second indication is only a hypothetic one, it can be used to calculate the AnTC of the second indication. The premium on the AnTC of the ACT can be calculated from the AnTC of the second indication and the AnTC of the ACT similarly to the cases with only one authorized indication. Both approaches have to be considered as an *approximation* of the actual negotiations. Which one is closer to the reality cannot be answered, since the negotiations are *confidential*. If the study designs are the same for both indications, the mixed calculations for the premiums can be used for the analysis. However, the mixed calculation approach is more condensed and it only provides an overall premium over both indications. Contrarily, the partial reimbursement amount approach considers different study designs in the various indications and offers results that are more meaningful; and is therefore the preferred one for this analysis.

For orphan drugs, not exceeding an annual revenue of 50 million Euro, no ACT is defined by the FJC and therefore no premium on an ACT can be derived. For orphan drugs, only a premium on comparable pharmaceuticals can be calculated and thus only for cases in competitive orphan indications. For this purpose, the AnTC of the comparable pharmaceuticals are weighted according to their market share. If for comparative pharmaceuticals with more than one indication the market share in the indication of interest is not referable, the mean of the AnTC of the comparable pharmaceuticals is used. For orphan drugs with an absolute soloist status, the negotiated reimbursement amount reflects the direct willingness to pay of the health care system.

For the difference between the premiums of substitutive versus additive study designs non-parametric Mann-Whitney U-tests were planned, since due to the small number of cases for each category a normal distribution cannot be tested or assumed.

4. In step four, the budget impact of the appraised pharmaceuticals for the SHI is analysed. In general, the budget impact is always crucial for a payer. Furthermore, the FJC already refers to a *virtual* budget impact in its decision, even before price negotiations of the pharmaceutical companies with the National association of the SHI start, as it includes in its decision quantities (target populations) and AnTC per capita of the appraised pharmaceutical and their ACT. This budget impact is only virtual, as it is not considering any market penetration or uptake assuming a *complete substitution* of the ACT and comparable pharmaceuticals by the new pharmaceutical in the approved indication.^10^ The indirectly in the FJC decisions included necessary information for the budget impact deviate from the *international standards* [25]. They do not include e.g. costs for the treatment of adverse events or subsequent disease complications. For the budget impact analysis, the additive premiums on the weighted ACT are used on an overall target population basis for a period of only 1 year from a SHI perspective. The chosen period is kept short, as especially in oncology many new products enter the market yearly or receive an authorization for new indications in short time after their first market authorization.
5. Finally, in step 5 further price negotiation influencing factors according to the *framework agreement* [18] were analysed in several univariate and multivariate regression analyses. These comprised (i) the extend of added benefit, (ii) comparable pharmaceuticals, (iii) European prices, (iv) AnTC of the ACT, and (v) the size of the target population. In accordance with our research question, we included in our regression next to the aforementioned variables (vi) the *study designs* as well. All but one variable (European prices) were publicly available and included either in the submitted dossiers by manufacturers or IQWiG’s assessments or FJC’s appraisals and decisions. For the European prices, adjusted at *purchase power parity* and weighted by the respective country population according to the Eurostat database [26], we referred only to those countries, in which at the end of negotiations between pharmaceutical companies and the National Association of Statutory Health Insurance Funds the respective pharmaceuticals are already reimbursed (i.e. list prices are available)^11^. The prices were derived from publicly accessible databanks [27–31]. For the UK prices were not public available. Therefore, we gathered the respective prices via the German Union of research based pharmaceutical companies (vfa) which was able to offer the information by its UK partner institution, the Association of the British Pharmaceutical Industry (ABPI). The regression analyses included only subgroups with an added benefit to consider premiums on the AnTC of the ACT. Orphan drugs were excluded from the analyses, as no premiums on ACT are negotiated for them.^12^ The *distribution* of the dependent variable and the independent variables were considered in the regression analyses as well. Since we wanted to *explain* annual treatment costs and *not to predict* them, we abstained from developing a parsimonious regression model based on stepwise elimination. The OLS-regressions were performed with STATA 14 and validated with the data analysis tool of MS Excel.

## Results

### Data basis and study assignment

For all 55 completed cases in our analysis period data were gathered from the above-mentioned sources. Some cases had to be excluded for different reasons: for example, the market authorization for Sipuleucel T was meanwhile withdrawn. For the first appraisal of Vandetanib no reimbursement was available, since it underwent its early benefit assessment during the intended transition period to enable all involved stake-holders to successfully adapt to the new legislation, and the negotiations started only after the completion of this transition period, i.e. after its second appraisal. For Idelalisib and the three orphan drugs Pomalidomide, Siltuximab and Blinatumomab no negotiated reimbursement amounts were available, since the arbitration board decided on their reimbursement with its award. Carfilzomib was at the end of our analysis period still in arbitration proceeding and in the case of Panobinostat, as an orphan drug with competition, the only available comparable orphan drug was Pomalidomide with an arbitrated reimbursement amount. Hence, after exclusion of the before mentioned cases 47 remained for further analysis. Subsequently, they were assigned to the respective study designs (Table 2). Fourteen cases or subgroups were assigned to an additive study design (3 without added benefit, 8 with a palliative ACT (BSC) and an added benefit, 3 with a curative ACT and an added benefit); 17 cases to a substitutive design (one with no added benefit, 16 with an added benefit); 7 to an orphan drug with competition in their indications, and finally 4 orphan drugs were absolute soloists. In 5 cases no studies or no accepted study designs by the IQWiG^13^ were submitted. These cases cannot be taken into further consideration, since there are no usable data on the study design. ACT not market authorized in Germany led as well to a *rejection* by the IQWiG. Similarly, for 18 subgroups, no studies or not accepted study designs by the IQWiG were submitted and they were excluded as well from the analysis.^14^ Yet, their population size and the AnTC of their ACT were considered within the premium calculations, respectively.

**Table 2:** Assignment to study design

Additive study design			Substitutive study design		Orphan drug with competition	Orphan drug soloist
Added benefit not proven	Added benefit proven palliative ACT (BSC)	Added benefit proven curative ACT	Added benefit not proven	Added benefit proven	Added benefit proven by law	
Pertuzumab 1. Ind 2. SG Regorafenib new EBA Pertuzumab 2. Ind	Cabazitaxel 1. SG Ipilimumab 1. Ind Abiraterone 1. Ind Vandetanib new EBA Enzalutamid 1. Ind Regorafenib 1. Ind Radium-223- dichloride 2. SG Ruxolitinib new EBA	Aflibercept Pertuzumab 1. SG Nintedanib	Dabrafenib	Eribulin 1. SG Vemurafenib Axitinib 2. SG Crizotinib 1. SG Vemurafenib new EBA Afatinib 1. SG Trastuzumab 2. SG Ruxolitinib Afatinib new EBA Nivolumab 2. SG Pembrolizumab 2./3. SG Trametinib Abiraterone 2. Ind Eribulin 2. Ind Enzalutamide 2. Ind	Decitabin Bosutinib Ponatinib Cabozantinib Obinutuzumab Ibrutinib Lenvatinib	Ruxolitinib Brentuximab Vedotin Ramucirumab Olaparib

*EBA* Early Benefit Assessment, *Ind* Indication, *SG* Subgroup

### Hypotheses testing

#### Additive study design with no proven added benefit (hypothesis 1)

Only 3 cases were assignable to this category^15^ (Fig. 2). For SG2 of the first indication of Pertuzumab no discount on the AnTC of the ACT can be proven, as with regard to subgroups a differentiated look on this case is only possible, if for the subgroup without added benefit it is assumed that the AnTC do not exceed the AnTC of the ACT. Otherwise, an equation with two unknowns would have to be solved. Therefore, this subgroup cannot be considered to test the hypothesis. For the re-assessment of Regorafenib a calculation of any discounts is not possible, since this pharmaceutical was withdrawn (opt-out) from the German market and no reimbursement price was negotiated thereafter. For the second indication of Pertuzumab both approaches, the mixed calculation and the partial reimbursement were utilized. Due to some specific peculiarities of this case (subsequent consideration of *manufacturer rebates*, potential *staggered rebate*, strongly diverging AnTC due to a pre-surgical application for the second indication) the partial reimbursement amounts were not considered. Hence, the second indication of Pertuzumab is considered together with the first indication by applying the mixed-calculation approach. This results in a multiplicative premium of 2.184 and an additive premium of 65,831.51 €, but is driven solely by SG 1 of the first indication of Pertuzumab. As a consequence thereof, the mixed calculation approach does not allow an exact estimation of the impact of the second indication of Pertuzumab on its reimbursement negotiations.

To sum up, none of the 3 cases with an additive study design and no proven added benefit enables an empirically verified conclusion as to whether hypothesis 1 can be accepted. Consequently, we have to rely on the legislation^16^ supporting this hypothesis and to test it in future again, when more cases assignable to this category will be available.

#### Substitutive study design with no proven added benefit (hypothesis 2)

To test for hypothesis 2 (Fig. 2), the case of Dabrafenib has to be investigated. For Dabrafenib the calculated multiplicative premium was 0.9762 (discount) and the additive premium on the AnTC of the ACT -2219.35 €, respectively. This result support hypothesis 2, that in case of a not proven added benefit the AnTC of the appraised drug do not exceed the AnTC of the ACT. This corresponds to the German Social Code, as well.

#### Additive study design with added benefit (hypothesis 3)

To test for hypothesis 3 we analysed all cases with an additive study design and an added benefit dividing into sub-categories: (i) those cases with a *palliative* ACT (BSC) and (ii) those cases with a *curative* ACT.

**Fig. 3:**
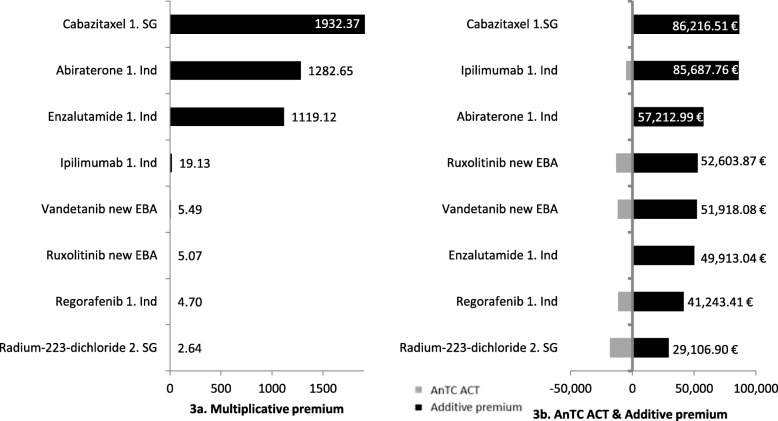
Additive study design with added benefit and BSC as ACT. Multiplicative and additive premiums are presented for cases with an additive study design with added benefit and best supportive care as appropriate comparative therapy

The multiplicative premiums of additive study designs with added benefit on palliative ACT (BSC) vary between 2.64 and 1932.37 (Fig. 3a). The highest premium was negotiated for Cabazitaxel, the lowest for Radium-223-dichloride, respectively. The lower the costs for BSC are, the higher the multiplicative premiums on BSC. We can conclude that the amount of AnTC of the ACT (BSC costs) has a strong influence on the extent of the (multiplicative) premium. Therefore, the differentiation between palliative and curative therapy seems reasonable, as the AnTC of palliative ACT are usually significantly lower than the AnTC of curative ACT. Even though low AnTC of ATC result in high premiums, it has to be expected that they will have a reducing effect on the total AnTC of the new therapy as price anchors. This effect will be checked in the subsequent regression analyses. The results for the additive premiums are presented in Fig. 3b. Again, Cabazitaxel with an added premium of 86,216.51 €, and Radium-223-dichloride with an added premium of 29,106.90, result in extreme values. Interestingly, Cabazitaxel shows a higher added premium than Abiraterone, even it was granted a lower added benefit (minor versus considerable) and both pharmaceuticals have exactly the same ACT, study design, target population and comparable pharmaceuticals in their indication (mCRPC).^17^ Further influencing factors on the premium have to be looked at for this specific case. The low multiplicative and additive premium for Radium-223-dichloride can be explained by the fact that in this case the manufacturer’s selling price (MSP) is taken into account (direct distribution to the nuclear medicine ambulatories) and thereby the reimbursement amount is lower.

**Fig. 4:**
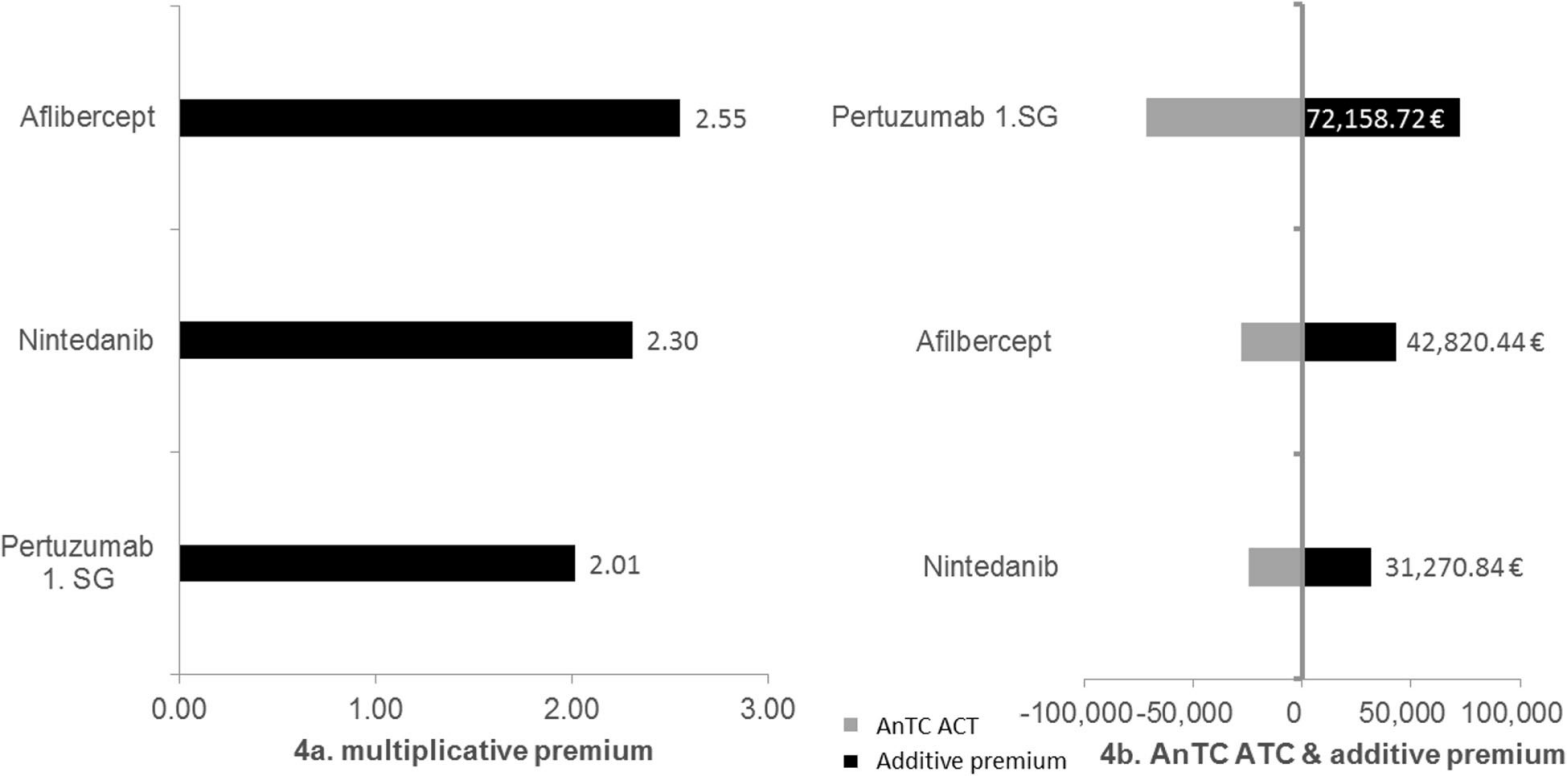
Additive study design with added benefit and curative ACT. Multiplicative and additive premiums are presented for cases with an additive study design with added benefit and a curative appropriate comparative therapy

Three cases were assigned to the category additive study design with added benefit and a curative ACT. Figure 4a shows that the multiplicative premiums in this category vary between 2.01 and 2.55 being, thus, much closer together than the premiums for the cases with a palliative ACT. The lower multiplicative premiums can be explained by the higher AnTC of the respective curative ACTs. The additive premiums for cases with additive study design and added benefit with a curative ACT reach values between 31,270.84 € and 72,158.72 € (Fig. 4b). Summarizing, all the cases with an additive study design (with a palliative as well as a curative ACT) and an added benefit result in a (multiplicative and additive) premium. Thus, hypothesis 3 can be confirmed.

#### Substitutive study design with added benefit (hypothesis 4)

Similarly when testing hypothesis 3, we calculated multiplicative premiums on the AnTC of the ACT for cases with a substitutive study design and an added benefit and thereafter the respective additive premiums. Unlike hypothesis 3, a differentiation in cases with a palliative and a curative ACT for the calculation of premiums is not necessary, since a complete substitution of the ACT with the new therapy is assumed, and therefore the budget effect is only the result of the difference of the costs for the new therapy and the costs of the substituted ACT without any surcharge on a basic therapy. Furthermore, no BSC was defined as an ACT in the case of substitutive designs. The multiplicative and additive premiums between additive and substitutive study designs were subsequently compared.

**Fig. 5:**
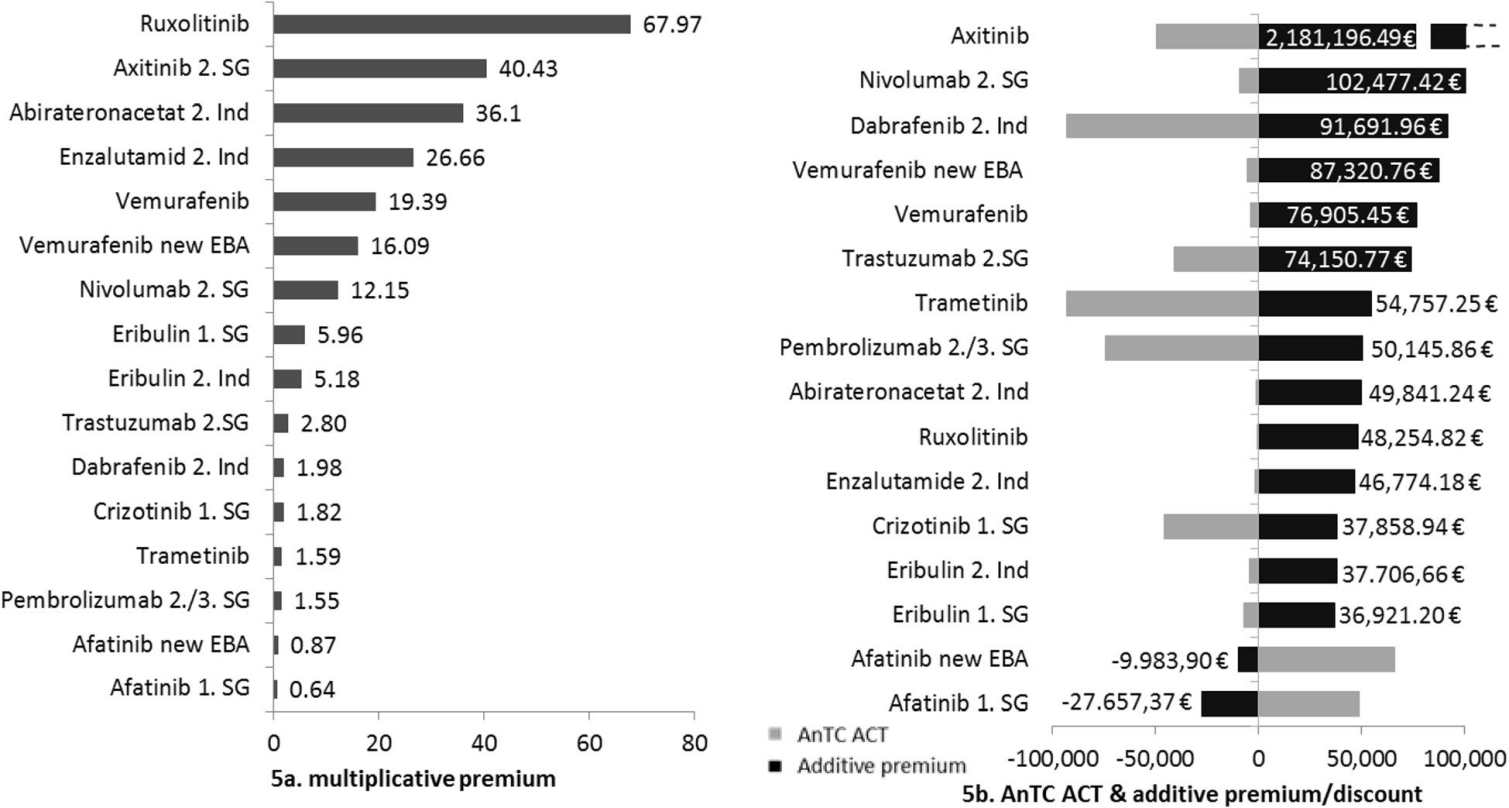
Substitutive Study design with multiplicative and additive premium. Multiplicative and additive premiums or discounts are presented for cases with a substitutive study design

The multiplicative premiums for a substitutive study designs with an added benefit varied between 0.64 and 67.97 (Fig. 5a). The highest multiplicative premium was achieved for Ruxolitinib. The lowest multiplicative premiums were those for Afatinib, which led to a discount even though an added benefit was granted to this pharmaceutical. This result can be explained by the price policy of the pharmaceutical company when the product entered the German market with an already lower price than the ACT. Due to the fact that no premiums are negotiated on the launch prices, the negotiated reimbursement amount lays below the AnTC of the ACT.

For the additive premiums in cases with a substitutive study design and an added benefit the order of pharmaceuticals change (Fig. 5b): The highest premium is reached by Axitinib. Yet, when applying the rule that values exceeding 3 standard deviations (SD = 516,813 €) are considered as outliers and therefore not included in the descriptive analysis. Axitinib^18^ has to be excluded. Afatinib on the other hand is again the pharmaceutical with the lowest premium for the reasons already explained above.^19^

The differences in the order of the analysed pharmaceuticals for substitutive study designs between multiplicative and additive premiums are a result of the higher impact of the AnTC of the ACT with regard to the multiplicative approach compared to the additive approach. The multiplicative approach is obviously more *sensitive* concerning extreme values.

Despite the differences between the two calculation-approaches both lead to the same overall conclusion: all cases except Afatinib with a substitutive study design and an added benefit show premiums on the AnTC of the ACT and support thereby hypothesis 4. Since the case of Afatinib is explainable by a specific condition, the hypothesis cannot be rejected.

**Fig. 6:**
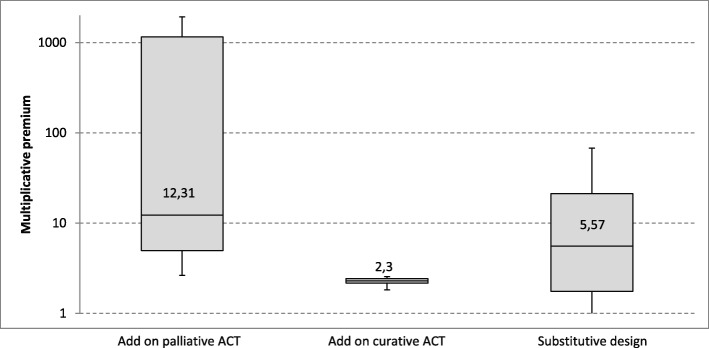
Multiplicative premiums depending on study design and appropriate comparative therapy. Multiplicative premiums are contrasted in box plots depending on study design and appropriate comparative therapy

In Fig. 6 the multiplicative premiums of the cases with an additive study design and a palliative ACT are compared with the respective premiums of the cases with an additive study design and a curative ACT as well as with premiums of those cases with a substitutive study design. In the presented Boxplots the y-axis is *logarithmic* to account for the high variance of the compared values. In case of an additive study design with palliative ACT the values (546.40 ± 728.33) vary much more than for additive designs with a curative ACT (2.29 ± 0.22) or substitutive designs (15.07 ± 18.54). Similarly to the mean values, the comparison of medians shows the lowest value for additive study designs with curative ACT (median = 2.3) followed by the median of substitutive study design (median = 5.57) and finally by the median of additive study designs with a palliative ACT (median = 12.31).

**Fig. 7:**
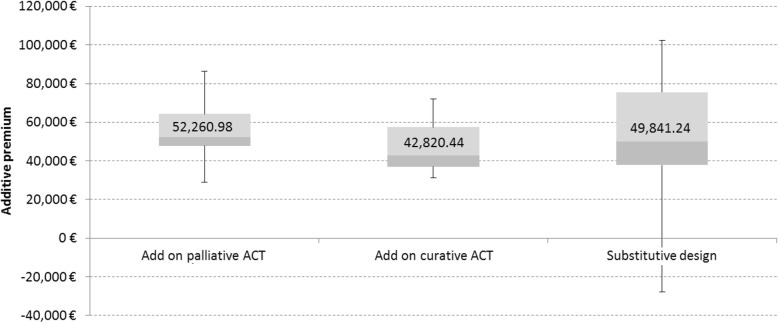
Additive premiums depending on study design and appropriate comparative therapy. Additive premiums are contrasted in box plots depending on study design and appropriate comparative therapy

As with the multiplicative premiums, differences of the additive premiums with regard to the study design occur as well (Fig. 7). Here, the observed variance is the highest for substitutive study designs (50,477.68 € ± 33.983.46 €) followed by the variance for additive study designs with palliative ACT (56,737.82 € ± 18,700.80 €) and the variance for additive study designs with curative ACT (48,750.00 € ± 17,210.94 €). Mean and median of the premiums for additive study designs with curative ACT (median = 42,820.44 €) are the lowest, followed by the values of substitutive study designs (median = 49,841.24 €) and additive study design with a palliative ACT (median = 52,260.98 €).

We abstained from the originally planned non-parametric Mann-Whitney U-tests with regard to the differences between the study design subgroups, since for that one with an additive design and a curative ACT only 3 cases were available and the test results would not be interpretable. Therefore, we presented the differences only *descriptively*.

Both medians and means of the multiplicative and additive premiums support hypotheses 3 and 4 particularly that the premiums are for substitutive study designs higher than for additive study designs. However, this only holds true for additive study designs with a curative ACT. For additive study designs with a palliative ACT both mean and median of the premiums are clearly higher than for substitutive study designs, leading at the first glance to a counterintuitive result, which is however explainable by the palliative ACT (BSC). This leads to the conclusion that cases with a palliative ACT should be looked into differently, since the palliative ACT are less costly and induce thereby high premiums. Hence, a common comparison of study designs with palliative and curative ACT is misleading with regard to their impact on the reimbursement amounts.

On the assumption that only additive study designs with a curative ACT are considered, hypothesis 4 can be confirmed, namely that in case of substitutive study designs the premiums are higher than those for additive study designs and vice versa hypothesis 3 can also be confirmed, namely that the premiums of additive study designs on the ACT are lower than in case of substitutive designs. Furthermore the results show that the study designs themselves are not the only relevant impact factor on the premiums and that other factors, like the choice of the ACT, play an important role as well. This will be further examined in the subsequent regression models.

#### Orphan drugs (hypothesis 5)

Hypothesis 5 postulates that analogous scenarios as in hypotheses 3 and 4 can be derived. In case of orphan drugs the premiums have to be calculated on the AnTC of comparable pharmaceuticals in the same indications, if any. For absolute *soloists* the reimbursement amount reflects the *absolute willingness to pay* of the SHI. Ruxolitinib, Pomalidomide and Ibrutinib exceeded the 50 million Euros annual revenue threshold and underwent a subsequent EBA like common pharmaceuticals. For the analysis period, negotiated reimbursement amounts for all Ruxolitinib appraisals, inclusively its new indication, were available as well, and therefore Ruxolitinib was assigned next to Orphan drugs to additive and substitutive study design. For Pomalidomide the arbitrated reimbursement amount was still applied after the new EBA. The new reimbursement amount for Ibrutinib could no longer be taken into account.

**Fig. 8:**
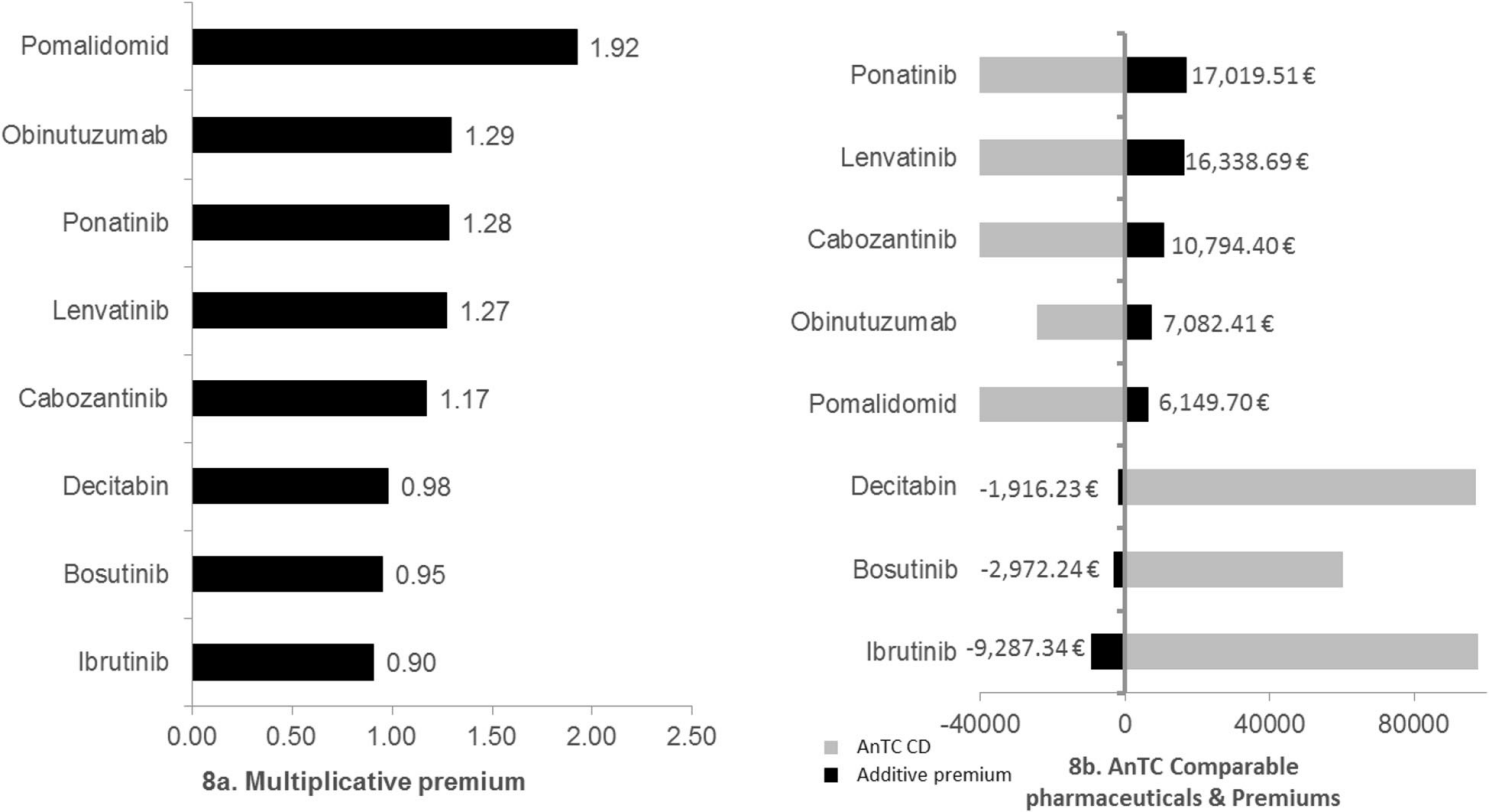
Multiplicative and additive premiums of orphan drugs with comparable drugs in their indication. Multiplicative and additive premiums of orphan drugs with comparable drugs in their indication are presented

With regard to the multiplicative premiums on the weighted AnTC of comparable pharmaceuticals there is a range between 0.90 and 1.92 (Fig. 8a). The mean (mean = 1.22) lays below the means of the premiums for the other study designs. Contrary to hypotheses 3 and 4, hypothesis 5 cannot be verified. The cases of Decitabine, Bosutinib and Ibrutinib show reimbursement amounts below the AnTC of their comparable pharmaceuticals. However, the discount is very small and might be the result of our own calculation of the weighted AnTC of comparable pharmaceuticals not including potential necessary other additional costs for the SHI. Another reason might be that the AnTC of comparable pharmaceuticals in orphan indications are higher than the AnTC of ACTs in the other categories. The same *pattern* can be seen when analysing the additive premiums (Fig. 8b).

**Fig. 9:**
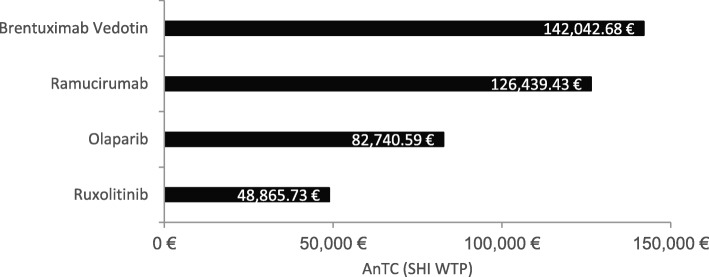
SHI Willingness to Pay for absolute soloist orphan drugs. The willingness to pay of the Statutory Health Insurance for absolute soloist orphan drugs is depicted

Figure 9 shows the AnTC of absolute soloist after negotiations varying for a myeloproliferative disease from 48,865.73 € up to 142,042.68 € for the therapy of Hodgkin lymphomas and reflecting the absolute willingness to pay of the SHI. The willingness to pay may depend on the budget impact of the orphan drug which depends in turn on the prevalence of the different orphan diseases.

In summary the primary hypothesis that the design of the submitted studies within early benefit assessment has an impact on the negotiated reimbursement amounts can be accepted. The same applies to the secondary hypotheses 2, 3 and 4 which strongly support the primary hypothesis. Secondary hypotheses 1 and 5 have to be further tested when more cases in these categories are available. Yet, they do not reject the primary hypothesis.

### Influence of study designs on the budget impact

**Fig. 10:**
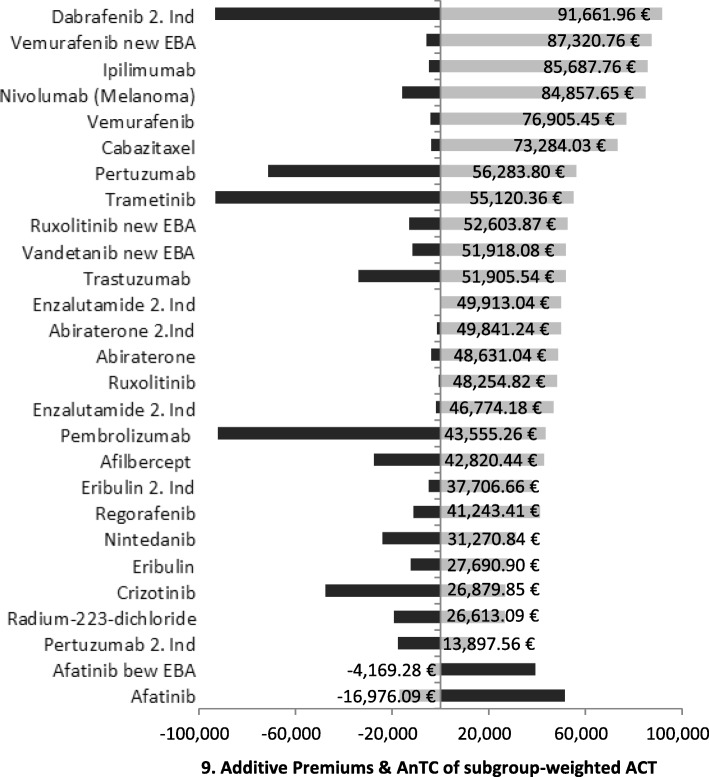
Additive Premiums & AnTC of subgroup-weighted ACT. The figure depicts the additive premiums in contrast to the annual therapeutic costs of subgroup-weighted appropriate comparative therapies

Since the study design of the assessed new oncology pharmaceuticals has an effect on the budget impact of the SHI, this effect will be analysed subsequently. Figure 10 shows the direct additional annual medication costs per capita for the SHI according to the additive premiums on the weighted ACT. The additive premiums on the weighted ACT vary considerably. For Afatinib they are even negative and in the case of the new indication of Dabrafenib additional costs per capita amount to 91,000 € per year.

**Fig. 11:**
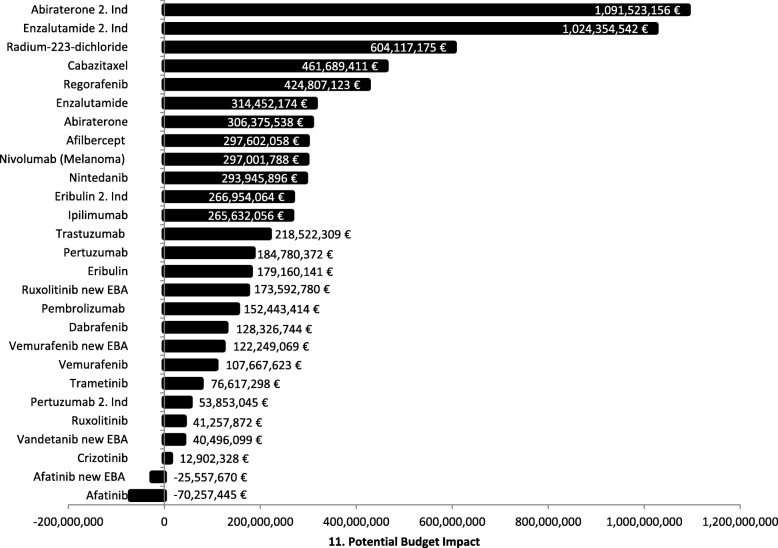
Potential Budget Impact. The figure shows the potential budget impact of new oncology products assuming a complete uptake of the products with regard to the whole target population, which is reflected by the respective prevalence of the subgroup-weighted indications

To estimate the additional total costs for the SHI, the additive premiums are multiplied with the target population size (Fig. 11). The target population size varies from approximately 400 up to 22,000 patients. We excluded orphan drugs from this analysis due to their very small target populations. Under the assumption that the total target population would be treated, the virtual additional budget impact for the SHI is for the new indication of Abiraterone with around 1 billion € the highest one. However, this budget impact will not be reached, since even in the case of absolute soloist, the uptake in the pharmaceutical market never reaches 100% and as long as there are comparable pharmaceuticals in the same indication available, i.e. prostate cancer in the case of Abiraterone, the market share is a matter of competition. Furthermore, the remaining life expectancy lays for some treated patients suffering from cancer below 1 year. Thus, the expected total annual medication costs for the SHI are not even reached in these cases.

The analysis of the budget impact for the SHI revealed that the size of the target population is the *main cost driver*. Therefore, the target population size will be examined next to other identified or statutory influencing factors on the premiums in the subsequent regression analyses.

### Regression analyses

The criteria contained in the framework agreement for the derivation of reimbursement amounts (extent of added benefit, comparable pharmaceuticals, EU-prices) [18] as well as further possible additive premium *influencing factors* like the amount of the AnTC of the ACT and the target population size are analyzed next to the study design within the OLS regression analyses. Only those cases were included in the regression analyses, for which a *complete data set* for all influencing factors were available. Cases with more than one indication were excluded, because the EU-prices were chronologically gathered only for the first indication. The *outlier* cases of Axitinib and Afatinib were excluded as well. This led finally to the inclusion of only 13 cases (see Supplementary Table regression input data). Table 3 shows the results for the univariate analyses of the influencing variables on the negotiated additive and multiplicative premiums.

**Table 3:** Results of univariate regression analyses

Intercept/Variable	Coefficient	Standard error	t-statistic	***p***-value
**Additive premiums**				
**EU prices (€)**				
Intercept	47,112.25243	5916.14299	7.96333904	0.00000682
X Variable	1.285149844	0.568349354	2.26119698	0.044997465*
**Comparable pharmaceuticals (€)**				
Intercept	48,424.19167	10,940.20482	4.426260061	0.001017904
X Variable	0.101908659	0.14325046	0.711401968	0.491646086
**Extent of added benefit (minor, considerable)**				
Intercept	35,653.9575	18,946.75333	1.881797735	0.086573666
X Variable	12,040.5225	11,230.66868	1.072110917	0.306628308
**AnTC of ACT (€)**				
Intercept	59,170.60488	7400.235335	7.995773405	0.00000657
X Variable	−0.155870503	0.186811151	−0.834374724	0.421820411
**Target population size (N)**				
Intercept	70,019.62133	7424.38089	9.43103841	0.00000132
X Variable	−2.195455811	0.862865137	−2.5443789	0.02726661*
**Study design (substitutive, additive)**				
Intercept	52,933.98625	7243.390467	7.307901803	0.00001527
X Variable	5642.11975	11,679.61618	0.483074072	0.63851445
**Study design (substitutive, additive curative ACT, additive BSC)**				
Intercept	36,397.2593	18,310.2111	1.98781211	0.07229695
X Variable	8106.2683	7572.83041	1.07044102	0.3073464
**Multiplicative premiums**				
**EU prices (€)**				
Intercept	314.560854	236.23716	1.33154688	0.20994402
X Variable	0.00380357	0.02269472	0.16759693	0.86994187
**Comparable pharmaceuticals (€)**				
Intercept	987.783989	290.778295	3.39703481	0.00596027
X Variable	−0.0099099	0.00380744	−2.6027873	0.02457156*
**Extent of added benefit (minor, considerable)**				
Intercept	473.047535	656.418807	0.72064897	0.48615854
X Variable	−83.468655	389.091577	−0.2145219	0.83406515
**AnTC of ACT (€)**				
Intercept	594.818068	223.036155	2.66691321	0.02191448
X Variable	− 0.0098356	0.00563031	−1.7468965	0.10847841
**Target population size (N)**				
Intercept	380.835803	308.693069	1.23370377	0.243024
X Variable	−0.0062737	0.03587646	− 0.1748683	0.86436022
**Study design (substitutive, additive)**				
Intercept	545.684925	219.766396	2.48302259	0.03041002
X Variable	− 539.42557	354.362665	−1.5222415	0.15616375
**Study design (substitutive, additive curative ACT, additive BSC)**				
Intercept	− 685.50644	547.170559	−1.2528204	0.23624936
X Variable	443.611998	226.301588	1.96026904	0.07577965

^*^p < 0.05 for X Variable

Despite the low number of cases, EU-prices and target population size became *statistically significant* in the *univariate regression* analyses for additive premiums (comparable pharmaceuticals for multiplicative premiums). The impact of both variables on the additive premiums was explored in a *bivariate regression* model with target population size remaining statistically significant (Table 4, model 4). After *log-transforming* the dependent variable additive premiums accounting for the variable’s distribution (Table 5), more than half of the *variance* could be explained by these two variables with the target population again remaining statistically significant (Table 4, model 5). To compare the *goodness-of-fit* for the calculated regression models containing differing numbers of transformed and untransformed independent variables we used the *adjusted r-squared*, adjusting for the number of terms in the model. Table 4 displays the regression models’ results in ascending order with regard to adjusted r-squared. Including the aforementioned independent variables in the OLS regression we started first for the additive premiums with a basis regression model using all variables untransformed (model 1 in Table 4). With an adjusted r-squared of 0.309, this model achieved only a fair goodness-of-fit. By log-transforming the dependent variable additive premiums and subsequently introducing an interaction term for AnT ACT and Target population, which reflects the *potential budget impact of the ACT*, adjusted r-squared could be improved stepwise 10% and additional 6%, respectively (model 2 and 3 in Table 4), reaching a moderate goodness-of-fit. After replacing the *dichotomized* study design (additive versus substitutive design) with a *trichotomized* operationalization, subdividing the additive designs in such with *curative* and such with *palliative* ACT, adjusted r-squared increased considerably (0.708) by additional 25% becoming now more substantial. When evolving stepwise the more *efficient* bivariate regression model with a moderate goodness-of-fit using only statistical significant variables by log-transforming the dependent variable additive premiums (model 5 Table 4) and subsequently additionally log-transforming the EU prices (model 6, Table 4), adjusted r-squared increased by 6% and additional 5%, respectively. Finally, by introducing to the bivariate regression model the study design with a trichotomized operationalization, adjusted r-squared reached with 0.610 an almost substantial goodness-of-fit (model 7, Table 4). Yet, the more efficient evolved bivariate model with fewer variables (statistical significant variables and trichtotomized study design) did not exceed the goodness-of-fit of the transformed basis regression model for the additive premiums. For both, the inclusion of study design as an independent variable was accompanied by a considerable increase of adjusted r-squared indicating that the study design itself can explain a *relevant proportion* of *variance*. Hence, it offers *valuable additional information* next to the target population size and AnTC, assuming that it is not *implicitly captured* by the *potential budget impact*. This is clearly shown by the increase of r-squared in model 8 (Table 4) for the basis regression model for additive premiums. Similarly, for the more efficient bivariate model the introduction of study design leads to an increase of adjusted r-squared (model 7, Table 4).

**Table 4:** Regression models with transformed and untransformed variables in ascending order of adjusted R-squared

Independent Variable	Coefficient	SE	95% CI	t statistic	***P*** value	R^**2**^	adj R^**2**^
**Additive premiums**							
**(1) Basis regression model using all variables untransformed, and additive premiums as the dependent variable**							
EU Prices	1.3141259	1.00407225	−1.1427504 – 3.77100219	1.30879616	0.23849493	0.654	0.309
Comparable drugs	−0.1535998	0.23822837	−0.7365236 – 0.42932406	− 0.6447585	0.54293375		
Added benefit	9034.29827	10,716.7639	−17,188.678 – 35,257.275	0.84300618	0.4315413		
AnTC ACT	−0.0486107	0.28074618	−0.7355719 – 0.63835042	− 0.1731484	0.86822835		
Study design dichotomized	7356.39568	15,843.3671	−31,410.927 – 46,123.7184	0.46432022	0.65878822		
Target population	−1.6576633	1.20885101	−4.6156151 – 1.30028857	−1.3712718	0.21936371		
Constant	52,107.00377	20,198.79989	2682.320947–101,531.6866	2.579707906	0.04178586*		
**(2) Basis regression model using all variables untransformed, and log additive premiums as the dependent variable**							
EU Prices	0.00001931	0.00001709	−0.00002252 – 0.00006114	1.12939086	0.30185338	0.705	0.410
Comparable drugs	−0.00000257	0.00000406	−0.00001249 – 0.00000736	−0.63303948	0.55005850		
Added benefit	0.19608572	0.18245474	−0.25036495 – 0.64253639	1.07470883	0.32381185		
AnTC ACT	−0.00000076	0.00000478	−0.00001245 –0.00001094	−0.15849576	0.87926624		
Study design dichotomized	0.07529218	0.26973604	−0.58472812 – 0.73531249	0.27913283	0.78951472		
Target population	−0.00003937	0.00002058	−0.00008973 – 0.00001099	−1.91280535	0.10430143		
Constant	10.84596964	0.34388803	10.00450596–11.68743333	31.53924778	0.00000007*		
(3) Basis regression model with interaction AnTC ACT*Target population, and log additive premiums as the dependent variable							
EU Prices	0.00002490	0.00001697	−0.00001871 – 0.00006852	1.46787027	0.20206636	0.775	0.460
Comparable drugs	−0.00000580	0.00000467	−0.00001781 – 0.00000620	−1.24238121	0.26918568		
Added benefit	0.04432483	0.21293062	−0.50303075 – 0.59168041	0.20816559	0.84331349		
AnTC ACT	−0.00000367	0.00000514	−0.00001688 – 0.00000954	− 0.71446967	0.50689617		
Target population	−0.00013334	0.00007798	−0.00033380 – 0.00006712	−1.70982331	0.14799077		
AnTC ACT*Tar. pop	0.00000000	0.00000000	0.00000000–0.00000001	1.24538235	0.26817087		
Study design dichotomized	−0.04186251	0.27475003	−0.74812996 – 0.66440493	− 0.15236582	0.88485596		
Constant	11.71190494	0.76927118	9.73443041–13.68937947	15.22467656	0.00002217*		
**(4) Bivariate regression model using statistical significant variables, and additive premiums as the dependent variable**							
EU-prices	1.00815416	0.49637946	−0.09784821 – 2.11415654	2.031015043	0.069686298	0.554	0.465
Target population	−1.80975847	0.78477917	−3.55835544 – −0.06116149	−2.306073507	0.043799202*		
Constant	61,129.98695	7879.338538	43,573.72662788–78,686.24727887	7.7582638	0.00001539*		
**(5) Bivariate regression model using statistical significant variables, and log additive premiums as the dependent variable**							
EU-prices	0.00001578	0.00000860	−0.00000338 – 0.00003494	1.83493479	0.09639450	0.606	0.528
Target population	−0.00003976	0.00001360	−0.00007005 – − 0.00000946	−2.92431420	0.01518788*		
Constant	11.02820319	0.13649721	10.72406846–11.33233792	80.79435053	0.00000000*		
**(6) Bivariate Regression model using log EU-prices and target population, and log additive premiums as dependent variable**							
Log EU-prices	0.14680259	0.06653394	−0.00144427 – 0.29504945	2.20643159	0.05187319	0.646	0.575
Target population	−0.00003672	0.00001317	−0.00006606 – --0.00000738	−2.788982	0.0191532*		
Constant	9.90990728	0.57996394	8.61766708–11.20214748	17.08710928	0.00000001		
**(7) Multivariate regression model using statistical significant variables, study design, and log additive premiums as the dependent variable**							
EU-prices	0.00000695	0.00000928	−0.00001405 – 0.00002794	0.74851855	0.47325401	0.707	0.610
Target population	−0.00004912	0.00001344	−0.00007953 – − 0.00001870	−3.6531866	0.00529125*		
Study design trichotomized	0.18888016	0.10707861	−0.05334849 – 0.43110881	1.76393916	0.11156758		
Constant	10.71083426	0.21852857	10.21648828–11.20518023	49.01342718	0.00000000*		
(8) Basis regression model with interaction AnTC*target population, trichotomized study design, and log additive premiums as the dependent variable							
EU Prices	0.00001430	0.00001280	−0.00001859 – 0.00004719	1.11734172	0.31464184	0.878	0.708
Comparable drugs	−0.00000489	0.00000346	−0.00001379 – 0.00000400	−1.41398786	0.21649968		
Added benefit	−0.26610636	0.21675823	−0.82330113 – 0.29108840	−1.22766442	0.27421249		
AnTC ACT	−0.00000604	0.00000394	−0.00001616 – 0.00000409	−1.53330417	0.18577935		
Target population	−0.00019253	0.00005941	−0.00034526 – − 0.00003980	−3.24045751	0.02293760*		
AnTC ACT*Tar. pop	0.0000000029	0.00000000	0.00000000–0.00000001	2.50391081	0.05423011*		
Study design trichotomized	0.29015387	0.13996625	−0.06964081 – 0.64994856	2.07302748	0.09288142		
Constant	11.81575737	0.51914558	10.48125116–13.15026357	22.76000749	0.00000304*		
**Multiplicative premiums**							
**(9) Basis regression model using all variables untransformed, and multiplicative premiums as the dependent variable**							
EU Prices	0.01863379	0.03046329	−0.0559072 – 0.09317478	0.61167992	0.56319335	0.708	0.417
Comparable drugs	−0.0170363	0.00722779	−0.0347221 – 0.00064944	−2.3570588	0.05651068		
Added benefit	318.494844	325.143866	−477.10353 – 1114.09322	0.97955052	0.36513746		
AnTC ACT	0.00768265	0.00851777	−0.0131596 – 0.02852487	0.90195532	0.40183525		
Study design dichotomized	−606.94617	480.68369	− 1783.1368 – 569.244448	−1.2626727	0.25356019		
Target population	−0.0694163	0.03667623	−0.1591598 – 0.02032722	−1.8926775	0.10725321		
Constant	1329.140422	612.8264012	−170.39176 – 2828.67261	2.168869389	0.073175976		
**(10) Basis regression model using all variables untransformed, and log multiplicative premiums as the dependent variable**							
EU Prices	0.00004607	0.00009979	0.12277548–9.94718239	0.46161051	0.66062263	0.815	0.630
Comparable drugs	−0.00005089	0.00002368	−0.00019812 – 0.00029025	−2.14954836	0.07515311		
Added benefit	2.23125516	1.06511407	−0.00010883 – 0.00000704	2.09485090	0.08105271		
AnTC ACT	−0.00000966	0.00002790	−0.37498507 – 4.83749539	−0.34604099	0.74112304		
Study design dichotomized	−2.19341764	1.57463515	−0.00007793 – 0.00005862	−1.39296880	0.21305128		
Target population	−0.00025771	0.00012014	−6.04641104 – 1.65957577	−2.14498038	0.07562852		
Constant	5.03497894	2.00751141	−0.00055169 – 0.00003628	2.50806990	0.04602317*		
**(11) Basis regression model using all variables untransformed, trichotomized study design, and log multiplicative premiums as the dependent variable**							
EU Prices	0.00002793	0.00010268	−0.00022332 – 0.00027919	0.27202759	0.79471928	0.822	0.646
Comparable drugs	−0.00004310	0.00002491	−0.00010406 – 0.00001786	−1.72989859	0.13437373		
Added benefit	0.81020226	1.23677559	−2.21607858 – 3.83648311	0.65509238	0.53669959		
AnTC ACT	−0.00001518	0.00002732	−0.00008202 – 0.00005167	− 0.55547716	0.59865283		
Study design trichotomized	1.51393827	1.00119466	−0.93589681 – 3.96377335	1.51213179	0.18125773		
Target population	−0.00021019	0.00010064	−0.00045646 – 0.00003607	−2.08848182	0.08176979		
Constant	2.41624100	2.26119548	−3.11670503 – 7.94918702	1.06856794	0.32635841		
**(12) Univariate regression model using log AnTC ACT, and log multiplicative premiums as the dependent variable**							
Log AnTC ACT	−0.931522444	0.036401089	−1.0116407 – − 0.8514042	−25.59051002	0.000000000037*	0.983	0.981
Constant	10.68460638	0.326107398	9.96684884–11.4023639	32.76407232	0.000000000003*		
**(13) Univariate regression model using log AnTC ACT, trichotomized study design, and log multiplicative premiums as the dependent variable**							
Log AnTC ACT	−0.9010848	0.04282724	−0.9965099 – − 0.8056598	−21.03999339	0.0000000013*	0.985	0.983
Study design trichotomized	0.2069238	0.16343973	−0.1572426 – 0.57109022	1.266055713	0.234191404		
Constant	9.947609983	0.663086674	8.4701608–11.4250592	15.00197541	0.0000000349*		

*adj* adjusted, *AnTC ACT* Annual therapeutic costs of appropriate comparative therapy, *CI* confidence interval, *SE* standard error

* ≤ 0.05

**Table 5:** Tests for normality

Dependent Variable	Shapiro-Wilk Statistic W	Critical value of W^**a**^	P-value
Additive premiums	0.918318	0.868535	0.238
Multiplicative premiums	0.584712	0.868535	0.000048*
**Transformed dependent variable**			
Log additive premiums	0.945428	0.868535	0.531
Log multiplicative premiums	0.762327	0.868535	0.0025*
**Metric independent Variable**			
EU Prices	0.588874	0.868535	0.000053*
Comparable drugs	0.907858	0.868535	0.171
AnTC ACT	0.817607	0.868535	0.011
Target population	0.772696	0.868535	0.0033*

^a^on a 5% significance level

^*^p ≤ 0.05

For the multiplicative premiums the basis regression including all variables untransformed (model 9, Table 4) lead to adjusted r-squared of 0.417 exceeding that one for the respective model for additive premiums (r-squared 0.309). By *log transforming* the dependent variable to account for the *missing normality* of the distribution of multiplicative premiums (Table 5) the r-squared improved about 21% (model 10, Table 4) and when *trichotomizing* the study design by additionally only 1.6% reaching 0.646 (model 11, Table 4). When optimizing r-squared data-driven by transforming independent variables, the univariate log-log model 12 (Table 4) with log AnTC of the ACT reached a tremendous r-squared of 0.981 exemplarily, which could be only marginally improved when adding to this model the trichotomized study design (model 13, Table 4). However, in OLS regression analysis only the *transformation* of *metric dependent variable* for *approximation* of *normality* is *meaningful* and guarantees an interpretable back-transformation. With the log transformation of both premiums the Shapiro-Wilk Statistic improved considerably even though for the log-transformed multiplicative premiums the null hypothesis had to be rejected as the calculated Shapiro-Wilk statistic W was less than the critical value of W (Table 5). Though transformation of independent variables in small samples to account for their distribution is mathematically possible, it results only in *artificial mathematical relations* violating the *central limit theorem*. Hence, the interpretable model with the best goodness-of-fit is for the additive premiums the basis regression model with the interaction AnTC*target population, trichotomized study design, and log additive premiums as the dependent variable (r-squared 0.708) and for the multiplicative premiums the basis regression model with trichotomized study design, and log multiplicative premiums as the dependent variable (r-squared 0.646). Both models explain a substantial part of variance, even though almost no independent variable became statistically significant due to the small number of observations.

## Discussion

The ‘Act to Reorganize the Pharmaceutical Market in the Statutory Health Insurance System’ was established in 2011 to reimburse new pharmaceuticals according to their added therapeutic benefit. With regard to the framework agreement when negotiating the reimbursement amounts the extent of the added benefit in comparison with an ACT, the comparable pharmaceuticals in the indication and the EU-prices have to be taken into consideration. Our analysis tested if the study design has next to the mentioned criteria of the framework agreement any impact on the negotiated reimbursement amounts. With the term study designs, we refer explicitly to the way the intervention arm is implemented in comparison to the control arm of the study. Other important factors with regard to design in oncology studies like endpoint operationalization [15, 33], study duration or cross-over [34] are captured by the granted added benefit.

The study design showed an impact on the premiums on the AnTC of the ACTs, both in the additive and multiplicative approach. Medians and means of the premiums were higher for substitutive study designs compared to additive study designs with a curative ACT. This could be shown in the regression analysis as well. The *signs* of the *explanatory variables* in the regression models were *plausible* and as anticipated. EU-prices were positive indicating that on a list price level the German prices are below the prices of the referenced European countries. This is in accordance with [35], who concluded that after the negotiations, Germany can be considered to be a relatively low-priced market compared with other EU countries. Furthermore, pharmaceutical companies strive for a fast market entrance in high-priced markets and therefore the first available EU-prices during the premium negotiations are relatively high. Comparable pharmaceuticals had a negative impact on the premiums reflecting the price structure in the specific oncology indications, which comprise next to innovative products older and generic products as well. The extent of *added benefit* (in the included cases considerable versus minor added benefit) had a *positive* impact on the premiums verifying the results of other publications [35–37]. The *annual therapeutic costs* of the *ACT* had a *negative* sign showing that the more expensive the comparator was, the less the negotiated premium on the comparator since it rises considerably the indication-specific price structure. Similarly, the *target population size* had a *negative* impact on the premiums, as it is part of the budget impact for the SHI.

*The primary hypothesis with regard to the impact of the study designs on the negotiated reimbursement amount is strongly supported*. However, the observed effect could have been more pregnant or within the regression analysis statistically significant. Yet, adding the study design to the explanatory variables, the adjusted r-squared increased in the interpretable models and, thereby, the amount of *explainable variance*. On the other hand, European prices, comparable pharmaceuticals, and target population size were in the univariate analyses *statistically significant explanatory variables*, with the latter remaining significant in some multivariate regressions. In accordance with the framework agreement [18], and contrarily to a recent publication [36], which used also a premium approach but a different regression, the size of the target population had a significant effect on the magnitude of premiums. With regard to European prices, Lauenroth and Stargardt 2017 showed also significant effects, but used a broader sample of European countries not accounting for the availability of their prices at the time of negotiations between pharmaceutical companies and the National Association of SHI.

Finally, the German health care system realized the importance and relevance of add-on study designs in oncology indications and an informal expert commission published a discussion paper on how to tackle with the *financial challenges* of the rather expensive combination therapies [38]. The authors of this discussion paper envisaged as possible solutions therapy advices released by the FJC, which should inform physicians about identified (free) combinations of *doubtful appropriateness*. Furthermore, FJC should introduce quality rules on use and prescription of these combinations in the outpatient health care, as this is the common praxis in inpatient health care [38]. The authors propose in case of free combination therapies, which exceed for instance a threshold of 10 million Euros annual sales *mandatorily* renegotiating the reimbursement of the respective oncology pharmaceuticals. This would lead to an additional *subsequent rebate* which could be abandoned in case the free combination receives a market authorization [38]. Nevertheless, negotiating add-on therapies for products of the same pharmaceutical company is a completely different situation in comparison with combination of products from different companies. Whereas in the first case the premium can be split for the combination products rather easily, in the second case a common negotiation with all the affected manufacturers would be necessary; this would probably raise some *antitrust concerns* in Germany. Furthermore, pharmaceutical companies developing and distributing monotherapies in oncology, which may serve unintendedly as combination partners, would be held liable for the actions of the manufacturers of the complementary combination products. The Union of the research-based pharmaceutical companies (vfa) has proposed to solve such kind of conflicts *confidentially* and *separated* for each company within *arbitration* to avoid antitrust concerns. Exemplarily, for Pertuzumab^20^ as an add-on intervention to Trastuzumab and Docetaxel in the indication of HER2-positive metastatic breast cancer with visceral metastases, with Pertuzumab and Trastuzumab being both products of Roche, only its premium was negotiated for this approved combination and the price of Trastuzumab was not affected. So far, no examples for an approved combination of two or more new oncology products from different pharmaceutical companies are available. Nevertheless, free and not approved combinations are definitely in current oncological health care applied, and therefore the above-mentioned discussion paper referred to them.

### Limitations

One reason for a missing statistical significance in the regression is the small number of cases for each category of study designs. We abstained from an inclusion of additional indications to increase the number of cases, as the other indications do not contain the *variability* in study designs compared to oncology pharmaceuticals. A higher number of cases with only substitutive study design would not be sufficient, since for each category of study designs more cases are needed to achieve a significant result. The number of cases is therefore predefined by the appraised oncology pharmaceuticals. Since there is an increasing trend for combination therapies in oncology, the number of additive study designs will rise in future and the results of the analysis can be validated with higher numbers of cases for the different study design categories. Furthermore, this will ease regression analyses since the explanatory power of their variables will grow.

Some of the assumptions made within the analysis may also have an influence regarding a less strong than probably expected effect of the study designs on the negotiated reimbursement amounts. Since the negotiations between pharmaceutical companies and the National Association of SHI Funds are *confidential*, detailed data on health services and costs are missing and, hence, *assumptions* have to be made for this data. Data on the *market share* in *real health care*, especially for the disaggregated level needed, are *not publicly* available and therefore the costs of a patient-individual therapy cannot be estimated exactly. The mean of all available therapies has then to be calculated and used as a proper *approximation*. Similarly, if a range for the patient target population size is given in the FJC decision, in absence of detailed health care data the mean of this range is again calculated and used for the analysis.

Likewise, costs are accompanied by *uncertainty* whenever cost ranges are given due to different dosages of the pharmaceuticals. We used for these cases again means of the ranges as the best *approximation*. Due to the fact that the FJC decision includes only minimal and maximal cost values, no calculations on a real cost distribution basis is possible. This would require additional data (SE, mode, alfa, beta etc.) for respective cost distributions (gamma, triangle, beta etc.). In many cases, costs for BSC are unknown as well. Hence, data published in the FJC decision, early benefit assessment and submitted dossier serve as relevant sources for BSC. Unfortunately, many times the FJC decision and early benefit assessment state for the BSC that their costs differ on a *patient-individual level*. In this case, respective cost data are derived from the submitted value dossier. Yet, it is possible that during the negotiations, updated or corrected data are presented and used, and thereby the negotiated premiums on the AnTC of BSC may somehow differ in regard to our own calculations.

Moreover, AnTC for the ACT within the reimbursement amount negotiations are considered on a daily basis. We used for our analysis the cost data from the FJC decision assuming that the prices would not change significantly in the 6 months after the publication of the FJC decision with the date of the publication of the negotiated reimbursement amount. Furthermore, the EU-prices referred to in the regression analyses are *list prices* not including *potential discounts* in the different European countries, which are kept often confidential.

Even the mixed-calculation approach and the partial reimbursement approach constitute *approximations* regarding the confidential negotiation content. This introduces some uncertainty as well.

All the *assumptions* made within the present analysis may be interpreted as limitations. Since *no transparent data* are available and the negotiation protocols are kept *strictly confidential*, the aforementioned limitations are inevitable. The attempt of our analysis is to simulate the confidential negotiation results to investigate the impact of the study design on the reimbursement amount. If access to health care and cost data is made possible in future, further analyses on the premiums on the AnTC of the ACT should be performed.

### Strengths

One the other hand, the strength of our analysis is the attempt, with the use of realistic assumptions and in absence of detailed data on health care and costs, to simulate the negotiations for the reimbursement amount. Moreover, contrary to other publications [39–42] we used in our analysis in accordance with the framework agreement the premiums on the AnTC of the ACT and not discounts on the list prices of the new pharmaceuticals when they entered the German market, as only a few recent publications have done as well [35–37]. The chosen *approach of a premium calculation* on the AnTC of the ACT is more complex, since the AnTC of the new therapies and the ACT have to be calculated. Especially for oncology pharmaceuticals, this is cumbersome and challenging due to patient-individual therapeutic schemes, which may depend on body size or surface. The calculation of discounts on a package basis after negotiations is definitely less complex. Nevertheless, the discount approach ignores pharmaceutical companies can take discounts to be rendered into account and thus adapt their list prices. This might bias the results leading to high discounts, as the calculation basis would be the list price and not the AnTC of the ACT, as intendent by the framework agreement.

In contrast to [36], who applied generalized linear model regression to analyse the impact of added benefit on the premiums and used an approximation by specifying a binary variable that captured whether comparable pharmaceuticals are available at the fourth level of the *Anatomical Therapeutic Chemical Classification* System, we quantified the target population weighted prices of comparable pharmaceuticals after identifying them either from the FJCs documentation of the definition of the ACT or after crosschecking the submitted dossiers by manufacturers or the respective clinical guidelines.

A further strength of the analysis is that contrary to other publications [35, 37] the premiums on the AnTC of the ACT are calculated on a *subgroup basis*. This is important, since in many cases with a different added benefit on a subgroup basis, the *subgroup-specific differentiation* of the premiums together with the respective subgroup sizes lead to *more valid results*, as convincingly shown for Axitinib with an added benefit granted only for one 1% of the labelled patient target population.

## Conclusion

The analysis of the impact of the study design on the negotiated premiums between pharmaceutical companies and payers showed that for oncology products with clinical studies following substitutive designs higher subgroup-specific premiums were achievable in comparison to those with an additive design and a curative comparator.

Hence, an *additional important influencing factor* of the negotiations next to those stated in the framework agreement was identified and verified. Therefore, study design should be considered by pharmaceutical companies and by decision makers and payers within strategic price planning as a potential predictor.

Pharmaceutical companies could anticipate the impact of the study design on the premiums when deciding upon the *clinical study program*. Whenever a substitutive design is implementable to replace the actual standard of care, higher premiums on the comparator in comparison to additive designs are achievable. However, it will not always be possible to follow substitutive designs, for example because of late or last line developments in oncology, which force for additive therapies or ethic commissions asking explicitly for an additive study design.

From a payer’s perspective, substitutive designs are in great demand, because the costs of new therapies are not added to those of the existing standard of care. The health care system already realized the *relevance* of *combination therapies* as *cost drivers*, especially in oncology, seeking for respective problem solutions to meet the financial challenges posed by them.

Further research is needed to determine the interaction between study design and the other influencing factors, in particular, when more oncology products will have been assessed and information on premiums can be derived on a more detailed basis.

It remains to be seen, whether an *algorithmic approach* of the negotiation will become apparent in the future. However, since negotiations are always based on *bargaining*, negotiating skills as a not quantifiable influencing factor should be also taken into account at least through the negotiation frequency as an indicator for *learning curve effects*.

## Supplementary information

**Additional file 1.** Table Input data regression models.

## Data Availability

The data for the regression models are included in a separate submitted supplement file. All the other data are publicly available and the respective sources are stated in the methods part and in the references.

## Abbreviations

ABPI: Association of the British Pharmaceutical Industry
ACT: Appropriate comparative therapy
AMNOG: Act to Reorganize the Pharmaceutical Market in the SHI System
AM-NutzenV: Legislative Decree on the benefit assessment of pharmaceuticals
AnTC: Annual therapeutic costs
BAH: German Association of Medicinal Product Manufacturers
BPI: German Pharmaceutical Industry Association
BSC: Best supportive care
CI: Confidence interval
EBA: Early benefit assessment
FJC: Federal Joint Committee
G-BA VerfO: Rules of procedure of the Federal Joint Committee
HRQoL: Health-related quality of life
Ind.: Indication
IQWiG: Institute for Quality and Efficiency in Health Care
mCRPC: Metastatic castration-resistant prostate cancer
MSP: Manufacturer’s selling price
ProGenerika: Association of the generic medicines manufacturers
PSP: Pharmacy selling price
SD: Standard deviation
SE: Standard error
SG: Subgroup(s)
SHI: Statutory health insurance
vfa: Union of the research-based pharmaceutical companies

## Footnotes

1 Points of criticism on IQWiG’s methods can be found in Röhmel [8], Witte and Greiner [9], Vach [10], Herpers and Dintsios [11].

2 For the observed variance in the added benefit reported by pharmaceutical companies, IQWiG, and the FJC see also Ruof et al. [16] and Peinemann and Labeit [17].

3 Until end of June 2016, the prices had to be weighted by the countries’ population.

4 The pharmaceutical companies are obliged by law to report the actual sales price, which is the retail price not including value-added tax minus the discounts that have been granted.

5 Mandatory manufacturer, wholesale and pharmacy rebates and their changes over time as well and new indications of the included pharmaceuticals were incorporated in the calculations, respectively. Up to the end of 2013, the mandatory rebate for manufacturers was 16%. On 1st January 2014 it was reduced to 6% and after 1st April 2014 it increased to 7%.

6 On a pharmacy sales price (PSP) basis.

7 The only exception was the price calculation for Radium-223-dichloride which is distributed due to its technology (alpha radiation emitter) direct to consumer (nuclear medicine ambulatory) and, therefore, the manufacturer sales price (MSP) was used for the calculation.

8 Par. 130b German Social Code V

9 Abiraterone, Eribulin, Regorafenib, Enzalutamide, Pertuzumab and Dabrafenib were approved for two indications, which were assessed in two different points of time.

10 Some data on market penetration of assessed pharmaceuticals is presented in de Millas et al. [24].

11 Austria, Denmark, Finland, the Netherlands, Sweden, and the UK.

12 For influencing factors on Orphan drug pricing in Germany see Schlander et al. [32].

13 One-armed studies (cohorts), not adjusted indirect comparisons or historical controls.

14 Cabazitaxel 2. SG, Eribulin 2. SG, Abiraterone 2. SG, Tegafur, Axitinib 1. SG, Crizotinib 2. SG, Pixantron, Pertuzumab 3. SG, Vismodegib 1. & 2. SG, Afatinib 2. & 3. SG, Ipilimumab new ind., Trastuzumab 1. SG & 3. SG, Eribulin 2. Ind. of 2. SG, Radium 223-dichloride 1. SG, Regorafenib new ind., Afatinib new ass. 2. & 3. SG, Nivolumab 1. SG & 3. SG, Pembrolizumab 1. SG, Trametinib 1. SG

15 Pertuzumab (first indication SG and second indication), and the re-assessment of Regorafenib after the termination of the time limitation of the first FJC.

16 Par. 130b sec. 3 German social code V

17 mCRPC: Metastatic castration-resistant prostate cancer.

18 The extreme value for Axitinib can be explained by the fact that the added benefit was granted only for one subgroup which refers to 1% of the labelled target population and which generates the respective premium.

19 One possible explanation for the price policy of the pharmaceutical company might be a very close launch price with regard to the comparable pharmaceuticals Gefitinib and Erlotinib for the indication of interest.

20 This was next to Nintedanib and Aflibercept one of the three presented cases with an add on design to a curative ACT.
