# Supplementary material for: The impact of additive or substitutive clinical study design on the negotiated reimbursement for oncology pharmaceuticals after early benefit assessment in Germany
Source: Health Econ Rev. 2020 Mar 14;10:7. doi: 10.1186/s13561-020-00263-2 (PMC7071579; doi:10.1186/s13561-020-00263-2)
Supplement: Supplementary file 1 — Additional file 1. Table Input data regression models. [file 13561_2020_263_MOESM1_ESM.docx]

Table Input data regression models

| Cases with  added benefit | Additive premium | EU-Prices | Comparable drugs | Extent of added benefit | Annual therapeutic costs of appropriate comparative therapy | Study design dichotomized | Target population | Study design trichotomized |
| --- | --- | --- | --- | --- | --- | --- | --- | --- |
| Cabazitaxel | 86,216.51 | 9,273.89 | 16,102.61 | 1 | 44.64 | 0 | 6,300 | 3 |
| Eribulin | 36,921.20 | 825.12 | 35,742.09 | 1 | 7,438.60 | 1 | 6,470 | 2 |
| Ipilimumab | 85,687.76 | 33,386.52 | 131,351.50 | 2 | 4,725.36 | 0 | 3,100 | 3 |
| Abiraterone | 57,212.99 | 3,429.26 | 19,183.17 | 2 | 44.64 | 0 | 6,300 | 3 |
| Vemurafenib | 76,905.45 | 4,228.49 | 90,413.12 | 2 | 4,180.30 | 1 | 1,400 | 2 |
| Afilbercept | 42,820.44 | 757.01 | 80,952.41 | 1 | 27586.52 | 0 | 6,950 | 1 |
| Enzalutamide | 49,913.04 | 6,854.79 | 25,769.30 | 2 | 44.64 | 0 | 6,300 | 3 |
| Trastuzumab Emtansin | 74,150.77 | 2,097.24 | 55,151.65 | 2 | 41132.87 | 1 | 4,210 | 2 |
| Nintedanib | 31,270.84 | 2,287.25 | 35,044.73 | 1 | 23973.40 | 0 | 9,400 | 1 |
| Pembrolizumab | 50,145.86 | 1,599.11 | 119,128.71 | 2 | 79868.07 | 1 | 3,500 | 2 |
| Trametinib | 54,757.25 | 5,638.79 | 126,325.63 | 2 | 93108.37 | 1 | 1,390 | 2 |
| Regorafenib | 41,243.41 | 9,189.92 | 61,212.47 | 1 | 11133.78 | 0 | 10,300 | 3 |
| Radium-223-dichloride | 29,106.90 | 1,273.88 | 55,737.93 | 2 | 45881.40 | 0 | 22,700 | 3 |
